# Tailoring the active site for the oxygen evolution reaction on a Pt electrode

**DOI:** 10.1038/s42004-022-00748-7

**Published:** 2022-10-13

**Authors:** Kazuki Iizuka, Tomoaki Kumeda, Kota Suzuki, Hiroo Tajiri, Osami Sakata, Nagahiro Hoshi, Masashi Nakamura

**Affiliations:** 1 Department of Applied Chemistry and Biotechnology, Graduate School of Engineering, Chiba University, Yayoi-cho 1-33, Inage-ku, Chiba, 263-8522 Japan; 2 Research and Utilization Division, Japan Synchrotron Radiation Research Institute (JASRI)/SPring-8, Kouto 1-1-1, Sayo-gun, Hyogo, 679-5198 Japan; 3 Synchrotron X-ray Group and Synchrotron X-ray Station at SPring-8, National Institute for Materials Science (NIMS), Kouto 1-1-1, Sayo-gun, Hyogo, 679-5148 Japan; 4 Center for Synchrotron Radiation Research, Japan Synchrotron Radiation Research Institute (JASRI)/SPring-8, Sayo-gun, Hyogo, 679-5198 Japan

**Keywords:** Electrocatalysis, Electrocatalysis

## Abstract

Highly active electrocatalysts for the oxygen evolution reaction (OER) are essential to improve the efficiency of water electrolysis. The properties of OER active sites on single-crystal Pt electrodes were examined herein. The OER is markedly enhanced by repeated oxidative and reductive potential cycles on the Pt(111) surface. The OER activity on Pt(111) is nine times higher in the third cycle than that before the potential cycles. OER activation by potential cycling depends on the (111) terrace width, with wider (111) terraces significantly enhancing the OER. The oxidation/reduction of the Pt(111) surface produces atomic-sized vacancies on the terraces that activate the OER. Structural analysis using X-ray diffraction reveals that the active sites formed by potential cycling are defects in the second subsurface Pt layer. Potential cycling induces the bowl-shaped roughening of the electrode surface, wherein high-coordination number Pt atoms at the bottom of the cavities activate the OER.

## Abstract (web summary)

Electrocatalytic oxygen evolution is a key reaction for water splitting, but the detailed atomic structures of single-crystal electrodes under cycling conditions are still not fully understood. Here, the authors study the oxygen evolution activity on Pt(111) during potential cycling and find that the current density reaches a maximum in the third cycle and is nine times higher than that in the initial cycle, owing to a roughened Pt(111) surface and formation of islands and atomic vacancies in the second subsurface Pt layer.

## Introduction

Hydrogen production using renewable energy and its subsequent utilization in fuel cells significantly contribute to a clean energy cycle^1,2^. Water electrolysis, which comprises the hydrogen evolution reaction (HER) and oxygen evolution reaction (OER) at the cathode and anode, respectively, is a useful method for hydrogen production. Typical electrolytes used commercially for water electrolysis include alkaline solutions and polymer electrolyte membranes (PEMs). Water electrolysis using PEM is advantageous owing to its operation at high temperatures and high current densities compared to alkaline solutions^3^. Although high HER activity has been achieved using noble metal electrocatalysts, the large overpotential of the OER causes significant energy loss. Under acidic conditions, electrocatalysts with enhanced corrosion tolerance are required to achieve high durability. Platinum group metals and their oxides, including IrO_2_ and RuO_2_, exhibit appreciable durability as anode materials in acidic media^4–6^.

Pt, which is active in several electrochemical reactions, is a widely studied noble metal in electrochemistry. Single-crystal Pt electrodes, characterized by a well-defined arrangement of surface atoms, enable active site identification for many electrochemical reactions^7–9^. Numerous surface science techniques have been applied to study interfacial structures encompassing the outer Helmholtz plane^10–15^. The scanning probe microscopy of atomically flat single-crystal surfaces can capture atomic motion in real-time, whereas X-ray diffraction can determine atomic coordination on the electrode surface.

Pt oxidation generates multiple oxidation states, including PtOH, PtO, and PtO_2_^16–20^. The presence of adsorbed OH and O at positive potentials can be detected using in situ vibrational spectroscopy, in situ X-ray absorption spectroscopy, and ex situ X-ray photoelectron spectroscopy^12,18–23^. However, oxidation of the electrode surface destroys the well-defined arrangement of surface atoms via place exchange with subsurface atoms. Even for noble metals, the electrode surface is highly oxidized at the positive potentials at which O_2_ is produced, resulting in the dissolution and/or restructuring of surface atoms^24–28^.

Although the formation of complex oxide layers, including α-PtO_2_ and β-PtO_2_, has been suggested at potentials at which the OER occurs^29^, few studies have investigated the detailed atomic structure of single-crystal electrodes under these conditions. Recently, scanning probe microscopy has been used to observe complex oxide layers at higher potentials^30–32^. Surface roughening through Pt oxide formation/reduction during potential cycling produces significant irregularities. X-ray diffraction also confirmed that irreversible oxidation is accompanied by the place exchange of Pt atoms in the subsurface layers^25,26,28^.

The electrode reactions are sensitive to the surface atomic arrangements. However, the atomic arrangements of single-crystal electrodes cannot be maintained at positive potentials resulting in subsurface oxidation. Recently, changes in the OER activity while holding the potential at 0.8 to 1.7 V vs RHE were investigated on Pt(111) and Pt(100) electrodes in an acidic solution^33^. OER activity decreased with increasing positive holding potentials owing to highly oxidized species formation. At negative holding potentials, the OER activity depends on the crystal orientation of the substrate, with Pt(100) being more active than Pt(111)^33^. This suggests that the atomic arrangement of the substrate affects the Pt oxide structure.

This study examines how the OER is influenced by the structural changes induced by electrochemical oxidation/reduction cycles on single-crystal Pt electrodes. The OER active sites is deduced using high-index planes with well-defined step-terrace structures. The electron density profiles, including the subsurface layer, are determined by measuring the X-ray crystal truncation rod (CTR). We find that the OER activity is sensitive to surface atomic defects produced by potential cycling.

## Results and discussion

### Dependence of OER activity on potential protocol

**Fig. 1:**
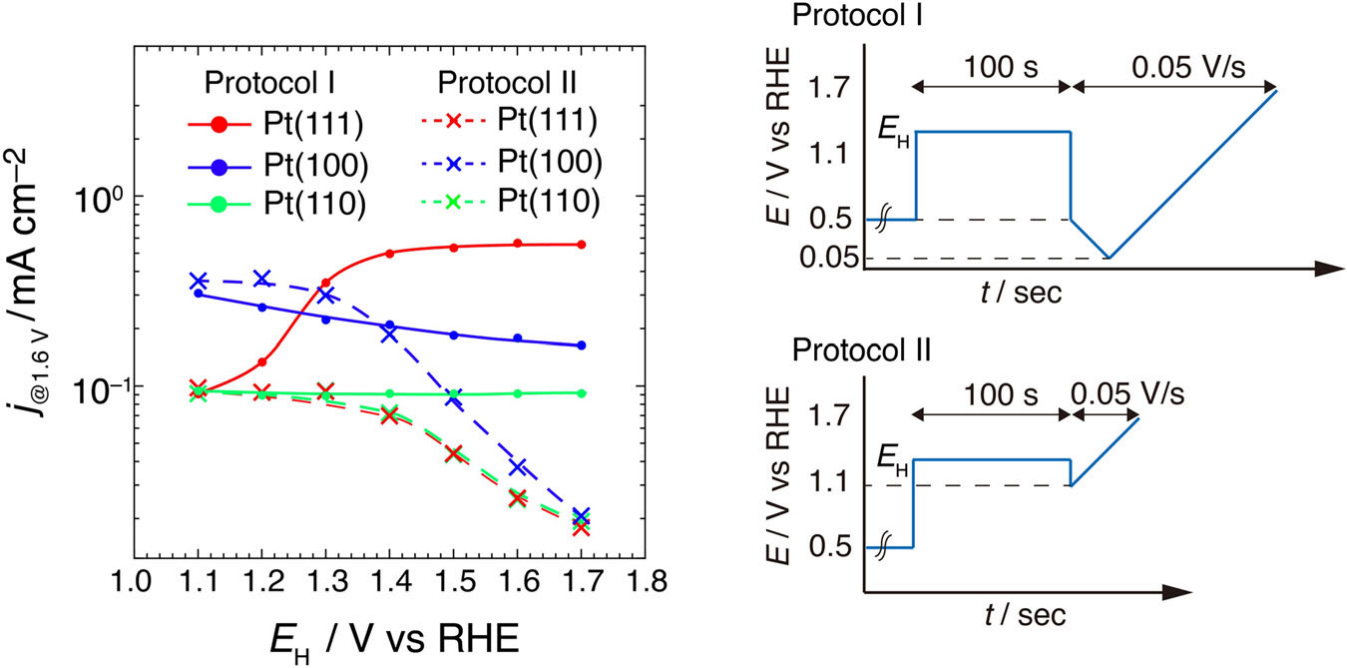
OER activity on low-index planes of Pt after two different potential protocols. Anodic current at 1.6 V on Pt(*hkl*) was estimated using potential protocols I and II in 0.1 M HClO_4_.

The specific adsorption of electrolyte ions affects the activity of electrochemical reactions^34,35^. The OER activity was evaluated in perchloric acid solution as it exerts only a small poisoning effect on the surface reaction. First, the OER activity of single-crystal Pt electrodes was investigated by maintaining the potential at a value where Pt oxide was formed. A previous study demonstrated that the crystal orientation of the Pt substrate affects the OER, with Pt(100) exhibiting higher activity than that Pt(111)^33^. The potential dependence of oxidation and reduction on the OER activity was tested using different potential protocols. Figure 1 shows the anodic current density of Pt(*hkl*) at 1.6 V (*j*_@1.6 V_) after holding the upper potential limit (*E*_H_) using two different potential protocols. After holding *E*_H_ between 1.1 and 1.7 V, the OER activity at 1.6 V was estimated using a potential scan from 0.05 V (protocol I) and 1.1 V (protocol II). The voltammograms of Pt(*hkl*) obtained with each protocol are shown in Fig. S1. For the potential protocol implemented previously (protocol II), the OER activity on Pt(100) is higher than that on Pt(111) and Pt(110), and a potential hold above 1.4 V decreases the activity on all low-index planes. The OER activities using the protocol without Pt oxide reduction are identical to those reported previously^33^.

The oxidation/reduction of the Pt surface causes the dissolution of surface Pt atoms and surface roughening, which may activate electrochemical reactions. Therefore, OER activity is estimated using a sequence involving the reduction of Pt oxide (protocol I). The OER activity is enhanced by a negative scan to 0.05 V after holding the potential above 1.4 V (protocol I). The *j*_@1.6 V_ on Pt(111) is six times higher than that on Pt(100), suggesting that the reduction of the higher oxidation states of Pt is required to enhance OER activity.

### OER activation on single-crystal Pt electrodes

**Fig. 2:**
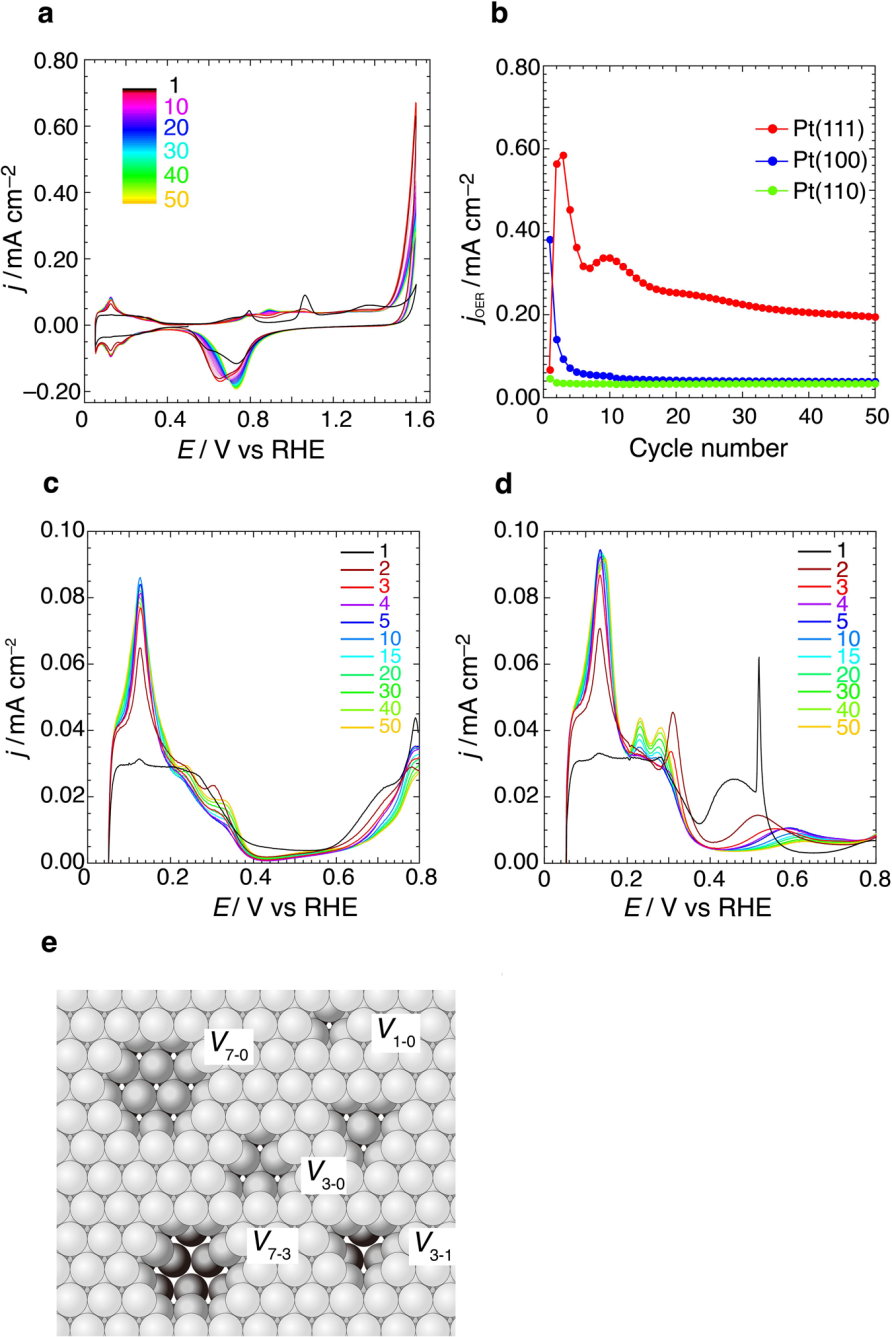
Potential cycle dependence of OER activity on low-index planes of Pt. **a** Cyclic voltammograms of Pt(111) in 0.1 M HClO_4_ between 0.05 and 1.6 V recorded during 50 consecutive potential cycles at a scan rate of 0.05 V s^–1^. **b** Potential cycle dependence of the specific OER activity (*j*_OER_) at 1.6 V on the low-index planes of Pt. **c**, **d** Enlargement of the Pt(111) voltammograms in 0.1 M HClO_4_ and 0.05 M H_2_SO_4_, respectively, between 0.05 and 1.6 V recorded during 50 consecutive potential cycles at a scanning rate of 0.05 V s^–1^. **e** Hard-sphere model of atomic size vacancies (single vacancy and 3–10 missing-atom vacancies), *Vx-y*, formed on the (111) facet, where *x* and *y* denote the number of extracted atoms in the topmost surface and the second subsurface layers, respectively.

As mentioned above, the reduction of Pt oxides is important for the activation of the OER. Here, the OER activity was investigated after the potential cycle, including the oxidation/reduction of the Pt surface. Figure 2a shows the cyclic voltammograms of Pt(111) in 0.1 M HClO_4_ upon potential cycling. In the positive segment of the initial potential scan, characteristic anodic peaks appear at 0.79 and 1.07 V^36^, with the anodic current increasing beyond 1.55 V owing to the OER. After potential reversal at 1.6 V and reduction of the initial oxidation products, the characteristic Pt(111) peaks disappear, and the hydrogen adsorption/desorption peaks at 0.13 V increase with continued cycling. The OER activity is significantly enhanced, and the onset potential of the OER shifts negatively by 0.1 V during the second and third potential cycles. After the fourth cycle, the OER current density at 1.6 V gradually decreases with continued cycling, and no significant changes in the hydrogen adsorption/desorption peaks at 0.13 V are observed.

The surface roughness of the electrode increases with continued redox cycling accompanied by a place exchange of subsurface Pt atoms^37^. The electrochemical surface area (ECSA) after each potential cycle in 0.1 M HClO_4_ was estimated using the charge density between 0.05 and 0.70 V in 0.05 M H_2_SO_4_. For Pt(111), the ECSA increases by approximately 1.2-fold from the 1st to the 50th cycle, as shown in Fig. S2. The specific OER activity normalized using ECSA, *j*_OER_, was estimated from the current density at 1.6 V after subtracting the background current originating from surface oxidation. Because the current density resulting from the surface oxidation depends on the potential cycle, the background currents were selected at a potential of 1.47 V for the 1st cycle and 1.35 V after the 2nd cycle, right before the onset of OER. Figure 2b shows the potential cycle dependence of the *j*_OER_ at 1.6 V on the low-index planes of Pt. On Pt(111), the *j*_OER_ value in the third scan is nine times higher than that in the first scan. Similar potential cycle measurements were performed on Pt(100) and Pt(110) surfaces, as shown in Fig. S3. Although *j*_OER_ exhibits the highest value during the initial scan on Pt(100), subsequent scans produce a sharp decrease in activity. Conversely, the *j*_OER_ on Pt(110) remains relatively unchanged throughout the potential cycling. The *j*_OER_ on Pt(111) is approximately five times higher than that on Pt(100) and Pt(110) after several tens of cycles. These results suggest that the oxidation/reduction cycles on the Pt(111) surface induce a unique structural change that activates the OER. The OER activity during the continued potential cycling also depends on the crystal orientations of the substrate, indicating that subsurface crystal orientation affects the surface structure even for fully oxidized Pt surfaces.

The voltammograms of the single-crystal Pt electrodes exhibit characteristic hydrogen adsorption/desorption shapes. Figure 2c and d show enlargements of the Pt(111) voltammograms obtained in 0.1 M HClO_4_ and 0.05 M H_2_SO_4_, respectively. A consecutive potential cycle was performed in a sulfuric acid solution because the voltammetric peaks are sensitive to surface defects and atomic orientation in this media. The cycle dependence of the OER activity in the sulfuric acid solution is identical to that observed in the perchloric acid solution (Fig. S4), indicating that the adsorption of (bi)sulfate anions does not affect the formation of OER active sites during potential cycling.

A characteristic redox peak appears at 0.31 V in the hydrogen adsorption/desorption region during the second and third potential cycles, wherein high OER activity was observed. Previous studies of Pt(111) in sulfuric acid solution show a similar peak appears after potential cycling up to 1.4 V^38,39^. In perchloric acid solution, the appearance of a small peak at 0.3 V was reported after the second potential cycle^32^ and was observed herein (Fig. 2c). This small peak originates from the single atom vacancy on roughened Pt(111) created by the place exchange of subsurface Pt atoms^32^. Although the imaging of the detailed atomic structure of small vacancies on a roughened surface is difficult using electrochemical scanning tunneling microscopy (EC–STM), the vacancy structure was estimated from the number of adatoms forming the average island shape^30^. These vacancies and islands on a (111) terrace contain the (111) and (100) steps. The effects of step structures on OER activity were investigated using the cyclic voltammetry of the Pt(331) and Pt(311) surfaces, which contain densely packed (111) and (100) steps, respectively. Anodic current densities at 1.6 V during the first cycle on Pt(331) and Pt(311) are comparable to those on Pt(111). However, the OER activity does not increase with increasing potential cycles (Fig. S5), indicating that the (111) and (100) step structures do not contribute to OER activation. Therefore, a site inside the vacancy may play an important role in OER activation. Figure 2e shows the stable models of the atomic size vacancies formed on the (111) surface. A previous EC–STM study suggested that the surface atomic vacancies form during the first few potential cycles and that the vacancy density gradually decreases with increasing cycles owing to the growth of vacancy/island structures^32^.

**Fig. 3:**
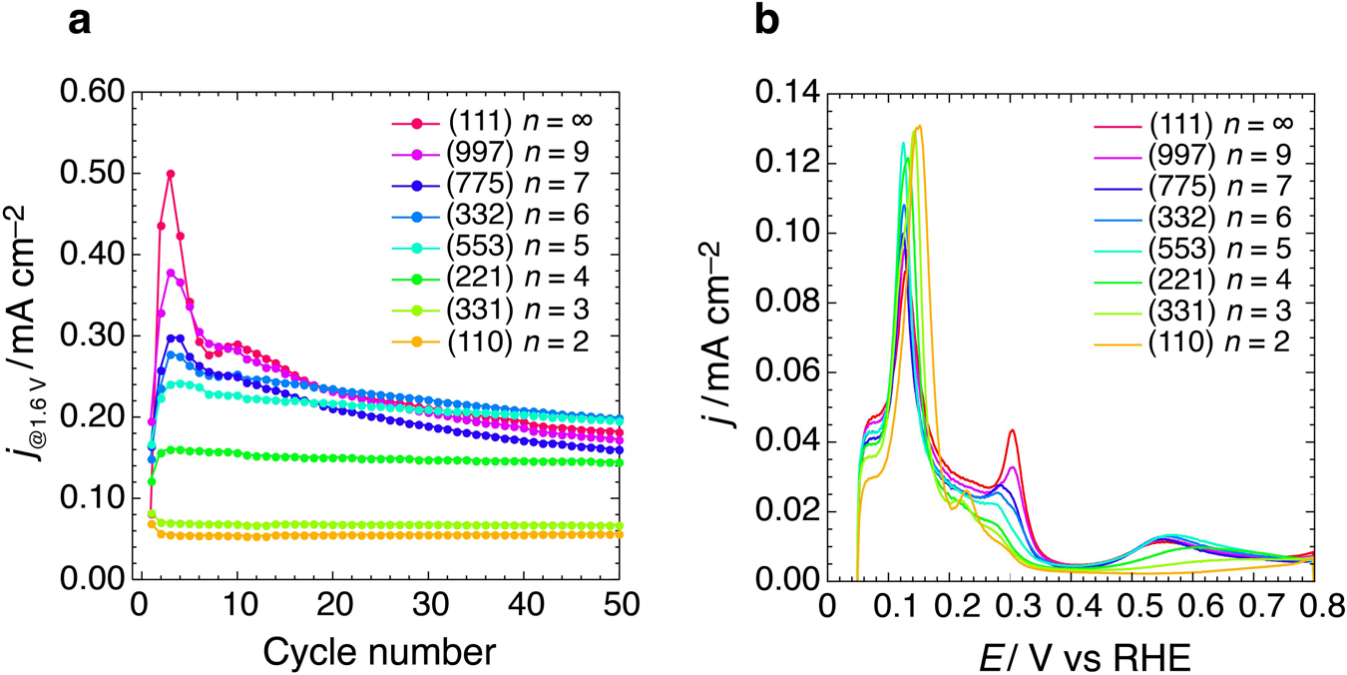
Potential cycle dependence of OER activity on high-index planes of Pt with (111) terrace. **a** Potential cycle dependence of the anodic current density at 1.6 V on the *n*(111)–(111) series of Pt. **b** Second-cycle voltammograms between 0.05 and 1.6 V of the *n*(111)–(111) Pt series (*n* = number of terrace atomic rows) in 0.05 M H_2_SO_4_ at 0.05 V s^–1^.

The OER behavior during continuous potential cycling on Pt(111) suggests that vacancy formation on a wide (111) terrace is essential for activation. The decrease in the OER activity after the fourth cycle can be attributed to decreasing number of effective vacancy sites. Increasing the number of potential cycles increases vacancy sizes and decreases their densities. Terrace vacancies created by subsurface oxidation cannot be readily characterized by a well-defined structure, such as a single-crystal surface. However, the vacancy size that yields OER activity can be estimated from the well-defined step-terrace structures of the single-crystal surfaces. We investigated the (111) terrace width necessary for vacancy-initiated OER activation using an *n*(111)–(111) series of Pt, where *n* is the number of terrace atomic rows. Figure 3a shows the potential cycle dependence of the anodic current density at 1.6 V (*j*_@1.6 V_) on *n*(111)–(111) Pt. The *j*_@1.6 V_ value in the third cycle increases with increasing (111) terrace width. Enhanced OER activity by successive potential cycling is observed on the surfaces wherein the terrace width is larger than five atomic rows. The vacancies that activate the OER require a wide (111) terrace with *n* > 5. On the *n*(111)–(111) with *n* > 5 surface, vacancies of various sizes (see Fig. 2e) can be created by the potential cycling. Although *V*_1_ vacancies will be formed on a (111) terrace with *n* > 3, the OER activity on Pt(221) with *n* = 4 is not initiated by successive potential cycles. The OER active sites are created via the oxidation of Pt atoms located inside atomic vacancies larger than *V*_3-0_. Figure 3b shows voltammograms for the *n*(111)–(111) Pt series in 0.05 M H_2_SO_4_ during the second potential cycle between 0.05 and 1.6 V. The peak and shoulder observed at 0.31 V appear only on the electrodes comprising terraces wider than the five atomic rows (*n* > 5). This indicates that vacancies larger than *V*_3-0_, which are formed on (111) terraces with *n* > 5, can activate the OER.

The potential cycle dependence of *j*_@1.6 V_ on *n*(111)–(111) indicates that a second local maximum at ~10 cycles is observed on the (111) terraces with *n* ≥ 7 (Fig. 3a). In the voltammogram on Pt(111), a new peak, known as “third hydrogen peak” at 0.23 V grows above 10 cycles (Fig. 2d). This third peak was assigned to the roughening of the (111) step on the (111) terrace in a previous study^40^. This suggests that the decrease in *j*_@1.6 V_ at the second maximum is related to the collapse of the (111) step.

### Structural determination of OER active site on Pt(111)

**Fig. 4:**
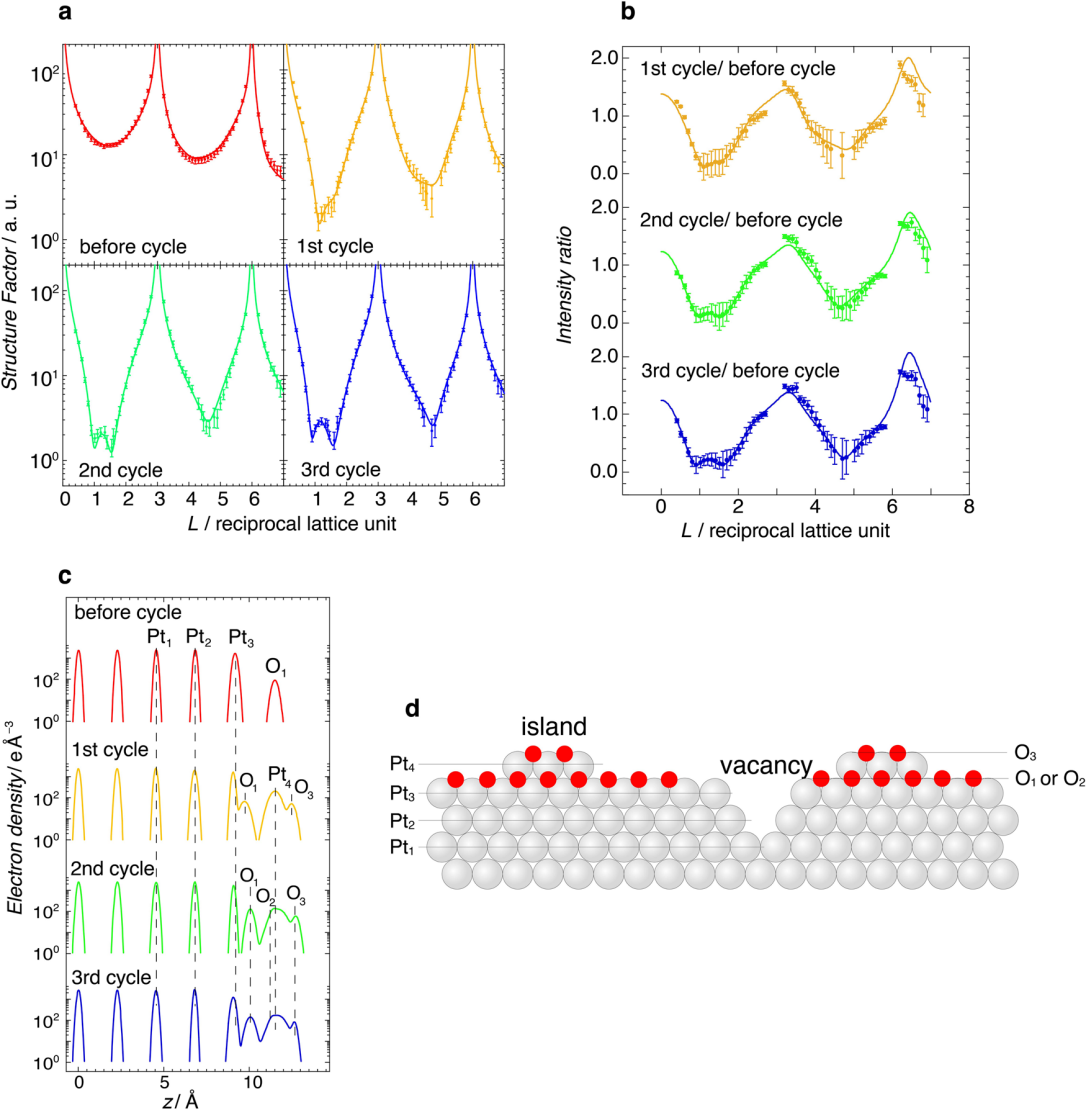
Structural determination of Pt(111) electrode after potential cycles using X-ray diffraction. **a** X-ray specular crystal truncation rod (CTR) of Pt(111) in 0.1 M HClO_4_ at 1.0 V after each potential cycle up to 1.6 V. X-ray specular CTR before the potential cycle was obtained at 0.9 V. **b** Specular CTR profiles normalized to the data at 0.9 V before the potential cycle. **c** Electron density profiles along the surface are normally optimized using specular and non-specular CTRs for each cycle. **d** Schematic diagram of the side view of the PtO layer on Pt(111). Pt and oxygen atoms are represented by gray and red spheres, respectively.

Although it is difficult to observe the detailed internal structure of atomic vacancies using EC–STM, electron density maps containing the subsurface can be determined using CTRs. The potential cycle dependence of the depth profile of the atomic density for atomic vacancies was evaluated from the occupancies (Occ) of the Pt layers. Figure 4a, b show the specular CTR of Pt(111) in 0.1 M HClO_4_ after each potential cycle and normalized CTR against the data at 0.9 V before the potential cycle, respectively. The non-specular CTRs used in the structural refinement are shown in Fig. S6. X-ray measurements were performed at 1.0 V following a scan to 1.6 V to prevent further oxidation caused by holding the potential at 1.6 V. Further oxidation or reduction of Pt oxide does not occur at 1.0 V. The CTR profile exhibits a symmetrical shape against the Bragg points before the potential cycling. After the second and third oxidations to 1.6 V, the scattered X-ray intensity decreases at *L* = 1.0–1.5 and 4.5–5.0, where scattering from the surface is dominant. This indicates that surface roughening occurs as a result of the oxidation/reduction cycles. The fine structure observed at approximately *L* = 1.0–1.5 is similar to that reported previously^16^ and clearly depends on the number of potential cycles. Structural optimization was performed using a model containing two PtO oxide layers and two subsurface Pt layers. Table 1 and S1 list the structural parameters and Debye–Waller (DW) factors of the optimized model, respectively. Figure 4c shows the electron density profiles along the surface normal for each scan. Models of Pt and O layers are illustrated in Fig. 4d.

**Table 1:** Vertical layer spacing (*d/*Å) and the occupancy factors (Occ) of oxygen and Pt layers estimated from X-ray CTRs at 1.0 V after surface oxidation until 1.6 V.

	before (0.9 V)	1st (1.0 V)	2nd (1.0 V)	3rd (1.0 V)
*d* (O_1_–Pt_3_)	2.34 ± 0.05	0.66 ± 0.06	0.99 ± 0.06	1.00 ± 0.05
*d* (O_3_–Pt_4_)		0.97 ± 0.09	1.02 ± 0.06	0.98 ± 0.07
*d* (O_1_–O_2_)			1.32 ± 0.08	1.26 ± 0.11
*d* (Pt_3_–Pt_4_)		2.46 ± 0.02	2.62 ± 0.02	2.60 ± 0.02
*d* (Pt_2_–Pt_3_)	2.33 ± 0.01	2.26 ± 0.01	2.27 ± 0.01	2.26 ± 0.01
*d* (Pt_1_–Pt_2_)	2.29 ± 0.01	2.27 ± 0.01	2.26 ± 0.01	2.26 ± 0.01
Occ O_3_		0.44 ± 0.04	0.39 ± 0.03	0.39 ± 0.04
Occ O_2_			0.35 ± 0.04	0.20 ± 0.03
Occ O_1_	0.69 ± 0.07	0.72 ± 0.07	0.92 ± 0.03	1.26 ± 0.05
Occ Pt_4_		0.28 ± 0.01	0.21 ± 0.01	0.28 ± 0.01
Occ Pt_3_	1.00 ± 0.01	0.72 ± 0.01	0.61 ± 0.01	0.63 ± 0.01
Occ Pt_2_	1.00 ± 0.01	1.00 ± 0.01	0.96 ± 0.01	0.94 ± 0.01
Occ Pt_1_	1.00 ± 0.01	1.00 ± 0.01	1.00 ± 0.01	1.00 ± 0.01
χ^2^	1.72	2.03	1.47	1.88

At 0.9 V before the potential cycle, the oxygen species, arising from the adsorbed hydroxide and water, are located at 2.34 Å from the Pt surface. The Pt layer spacings are identical to those reported previously^41^. The uppermost layer of the electrode is oxidized following the initial oxidation, and the spacing between the first and second Pt layers (*d*(Pt_3_–Pt_4_)) is expanded compared to that of bulk Pt. The occupancies of the first (Pt_4_) and second (Pt_3_) layers were determined to be 0.28 and 0.72, respectively, indicating that the topmost Pt atoms are partially lifted by place exchange. The occupancy of the place-exchanged Pt (Pt_4_) is less than that obtained using X-ray CTR measurements at a constant potential in the Pt oxidation region^42,43^. When the potential is held in the Pt oxidation region, surface roughening dynamically progresses^43^. Herein, the CTRs after the potential scan up to 1.6 V were measured at 1.0 V, where no further oxidation occurs to examine the OER active surface structure. Therefore, the occupancy of the place-exchanged Pt is lower than that of previous studies. The DW factors for Pt and O in the Pt oxide layers are larger than those in the inner Pt layer, indicating a disordered structure^25,44^. Especially the place-exchanged atoms (Pt_4_ and O_1_) have large in-plane DW factors^25,43^, and we cannot determine the in-plane site of the Pt oxide layer. The place-exchanged Pt atoms during the initial oxidation do not activate the OER.

After the second cycle of OER activation, the *d*(Pt_3_–Pt_4_) expanded further. Moreover, the occupancy of the subsurface Pt_2_ layer decreases from 1.00 to 0.96, suggesting that the defect sites are produced at the subsurface Pt_2_ layer in the vacancies. A roughened surface is formed from the irregularities caused by the presence of vacancies and islands. The number of Pt atoms in the Pt oxide layer after the second cycle is less than that observed at the initial oxidation, because the reduction of Pt oxides causes the dissolution of place-exchanged Pt atoms^44^.

The oxygen atoms of the O_1_ and O_3_ layers are bonded to the Pt atoms of the Pt_3_ and Pt_4_ layers, respectively. After the second cycle, the distance between the O_2_ and Pt_4_ layers got considerably smaller (not shown in Table 1). Previous density functional theory (DFT) calculations suggest that the oxygen atoms in the PtO_2_ layer exhibit a complex coordination structure with PtO_3_, PtO_4_, and PtO_5_ oxide units^45^. Although the Pt_4_ layer may form PtO_2_ containing O_3_ and O_2_, the ratio of Occ O_2_ + Occ O_3_ to Occ Pt_4_ is >2. However, the ratio of Occ O_1_ + Occ O_2_ + Occ O_3_ to Occ Pt_3_ + Occ Pt_4_ is ~2, indicating that the total surface oxide layer corresponds to a PtO_2_ composition.

Vacancies larger and deeper than *V*_3-0_, such as *V*_3-1_ and *V*_7-3_, must form defects in the subsurface Pt_2_ layer, as shown in Fig. 2e, consistent with the fact that surfaces with *n* < 5 do not activate the OER. X-ray structural analysis indicates that the layer spacing between subsurface Pt_2_ and Pt_3_ (*d* (Pt_2_–Pt_3_)) is comparable to that of bulk Pt. Therefore, the bottom of the cavity (the Pt_2_ layer) maintains its metallic state. The bottom Pt atom retains a large coordination number, resulting in lower binding energy for the adsorbed oxygen. Water oxidation reactivity may be enhanced at such sites.

Previous DFT calculations and surface X-ray studies have suggested the formation of specific stripe oxide by the extracted Pt on oxidized Pt(100)^28^. The dissolution rate of the surface Pt atoms on Pt(100) is one order of magnitude higher than that on Pt(111). The OER activity on Pt(100) decreases with an increasing number of potential cycles owing to the instability of surface Pt atoms and the absence of high coordination-number Pt sites.

## Conclusion

The OER was investigated on single-crystal Pt electrodes, showing significantly enhanced reactivity on Pt(111) owing to continuous potential cycling between 0.05 and 1.6 V vs RHE. The OER current density on Pt(111) reaches a maximum in the third cycle and is nine times higher than that in the initial cycle. Potential cycling roughened the Pt(111) surface owing to the formation of islands and atomic vacancies on the terrace, resulting in OER activation. The vacancy size required for OER activation was determined using the well-defined high-index planes of Pt with a step-terrace structure. X-ray CTR measurements revealed that defects in the second subsurface Pt layer activated the OER. The potential cycling of the Pt surface induced a bowl-shaped roughening, suggesting that the high coordination-number Pt atoms at the bottom of the cavities activated the OER.

## Methods

### Single-crystal Pt electrodes and materials

The single-crystal Pt beads used in the voltammetry experiments were prepared using Clavilier’s method^46^. The Pt(111) disk electrode used in X-ray measurements was purchased from Mateck (Germany). The electrolyte solutions were prepared in ultrapure water using Ultrapure H_2_SO_4_ (Kanto Chemical) and Ultrapure HClO_4_ (Kanto Chemical).

### Voltammetry

The samples were annealed using an H_2_/O_2_ flame. The samples were then cooled to room temperature, protected with ultrapure water, and transferred to an electrochemical cell. A reversible hydrogen electrode (RHE) was used as the reference electrode for all measurements. The potential cycles for activating the OER were initiated by scanning in the negative direction from 0.5 V and subsequently cycling between 0.05 and 1.60 V. The determination of the ECSA in perchloric acid solution was accomplished by transferring the electrode after a given number of potential cycles in 0.1 M HClO_4_ to a cell containing 0.05 M H_2_SO_4_ and measuring the charge density between 0.05 and 0.70 V.

### X-ray diffraction measurements

X-ray CTR measurements were performed using a multi-axis diffractometer at BL13XU (SPring−8)^47,48^ and BL3A (KEK PF). Specular and non-specular CTRs were measured using X-ray beam energies of 20 and 14 keV, respectively. A beam size of 100 μm × 100 μm was obtained using a four-quadrant slit. A drop cell was used, with the RHE reference and Au counter electrodes immersed in the electrolyte droplet on the surface^49^. The diffracted photons were counted using a Ce-doped yttrium aluminum perovskite (YAP:Ce) detector and a silicon drift detector. The CTR intensities were collected along the *L* direction normal to the surface, where the scattering vector, *Q*, for the reciprocal wave vector is defined as *Q* = Ha* + Kb* + Lc* (a* = b* = 4π/√3*a*, c* = 2π/√6*a*, where *a* is the nearest neighbor distance of the bulk Pt i.e., 2.775 Å). Integrated intensities were obtained by rocking scans and subsequently corrected for irradiated surface area, X-ray path length through the electrolyte solution and Lorentz factor. Structural optimization was performed using the least-squares method using the ANA − ROD program^50^.

The optimized model at 0.9 V before the potential cycle was composed of adsorbed oxygen layer (H_2_O_ad_ or OH_ad_) at the atop site and Pt layers. The models after surface oxidation until 1.6 V were composed of Pt oxide (Pt_4_, O_1_, O_2_, and O_3_) and subsurface Pt layers (Pt_1_, Pt_2_, and Pt_3_). For the Pt_4_ atom, we assumed the fcc and hcp sites with the same occupancy factor.

A total of 419–440 reflections along the specular and non-specular CTRs containing equivalent rods were collected for each cycle. The structure factors were averaged assuming a P3 space group and yielded 131–135 independent reflections that were used for the structural optimization. The symmetrical reproducibility of each cycle is presented in Table S1. The atomic coordinates, scale factor, occupancy factor, and anisotropic Debye–Waller factor of each atom were optimized using a (1 × 1) unit cell of the Pt(111) surface for the initial model. The agreement between the optimized model and experimental data was estimated using the goodness of fit χ^2^.

## Supplementary information

Supplementary Information

Description of Additional Supplementary Data

Supplementary Data1

Supplementary Data2

Supplementary Data3

Supplementary Data4

## Data Availability

The data that support the findings of this study are available within the paper and Supplementary Information, as well as from the corresponding author upon request. Numerical data for each figure are available in Supplementary Data 1, 2, 3 and 4.
