# Supplementary material for: Tailoring the active site for the oxygen evolution reaction on a Pt electrode
Source: Commun Chem. 2022 Oct 13;5:126. doi: 10.1038/s42004-022-00748-7 (PMC9814662; doi:10.1038/s42004-022-00748-7)
Supplement: Supplementary file 1 — Supplementary Information [file 42004_2022_748_MOESM1_ESM.pdf]

Tailoring the Active Site for the Oxygen Evolution Reaction on a Pt Electrode

Kazuki Iizuka,[†] Tomoaki Kumeda,[†] Kota Suzuki,[†] Hiroo Tajiri,[‡] Osami Sakata,[§¶] Nagahiro Hoshi,[†] Masashi Nakamura*[†]

[†] Department of Applied Chemistry and Biotechnology, Graduate School of Engineering, Chiba University, Yayoi-cho 1-33, Inage-ku, Chiba 263-8522, Japan.

[‡] Research and Utilization Division, Japan Synchrotron Radiation Research Institute/SPring-8, Kouto 1-1-1, Sayo-gun, Hyogo 679-5198, Japan.

§ Synchrotron X-ray Group and Synchrotron X-ray Station at SPring-8, National Institute for Materials Science (NIMS), Kouto 1-1-1, Sayo-gun, Hyogo 679-5148, Japan.

¶ Center for Synchrotron Radiation Research, Japan Synchrotron Radiation Research Institute (JASRI), Sayo-gun, Hyogo 679-5198, Japan.

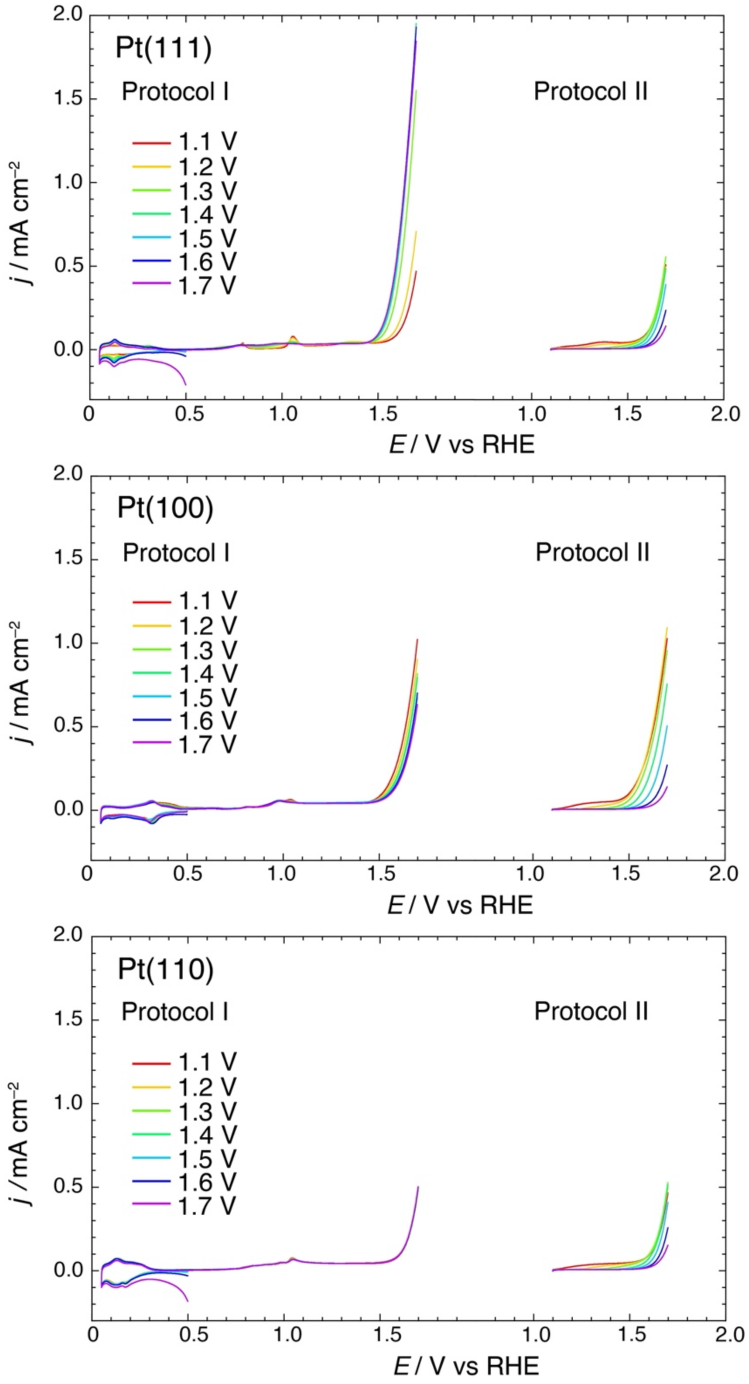

Figure S1. Voltammograms of the low index planes of Pt in 0.1 M $HClO_4$ recorded at scanning rate of 0.05 V s^{-1}, implementing potential protocols I and II.

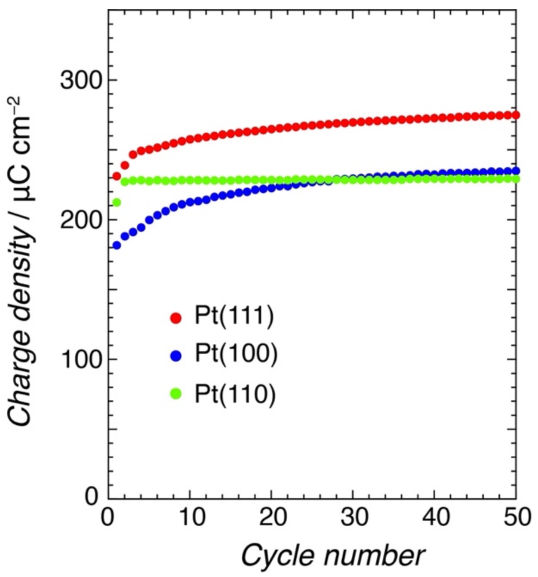

Figure S2. Electrochemical surface area (ECSA) of the low-index planes of Pt estimated from the charge density between 0.05 V and 0.70 V in 0.05 M H_2SO_4 after successive potential cycles in 0.1 M $HClO_4$.

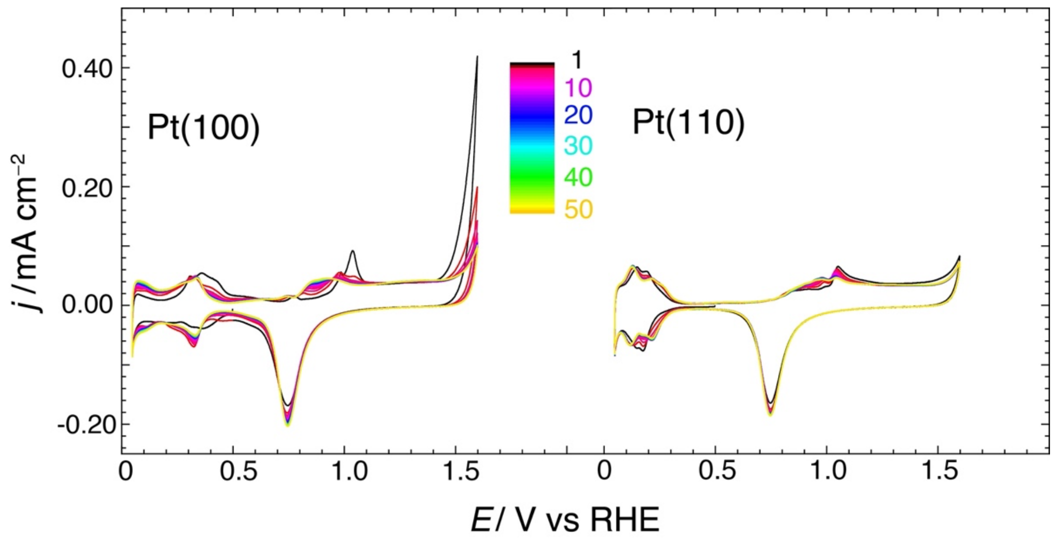

Figure S3. Cyclic voltammograms of Pt(100) and Pt(110) in 0.1 M $HClO_4$ between 0.05 and 1.6 V recorded over 50 potential cycles at scanning rate of 0.05 V s^{-1}.

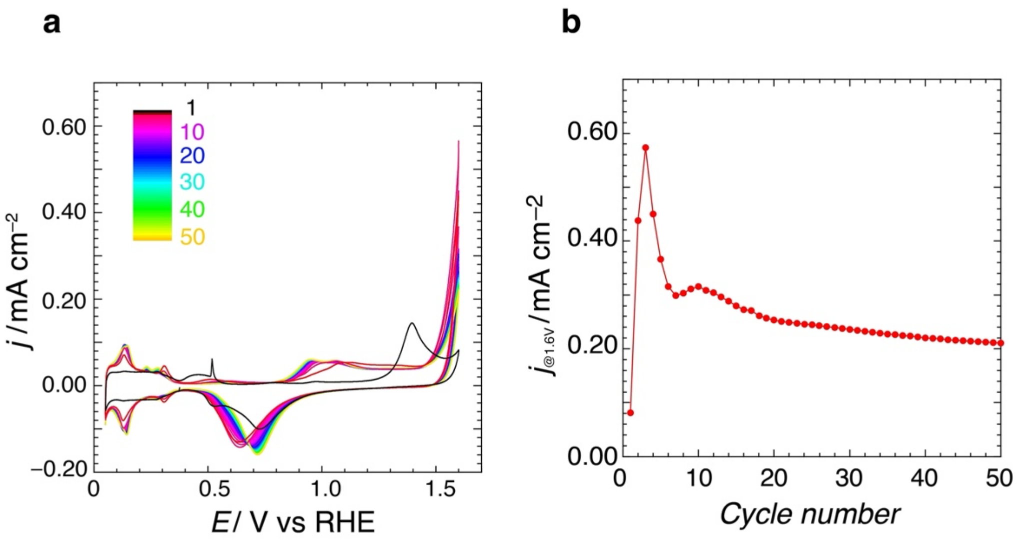

Figure S4. a, Cyclic voltammograms of Pt(111) in 0.1 M H_2SO_4 between 0.05 and 1.6 V recorded over 50 potential cycles at scanning rate of 0.05 V s^{-1}. **b**, Potential cycle dependence of the OER current densityat 1.6 V on Pt(111) in 0.1 M H_2SO_4.

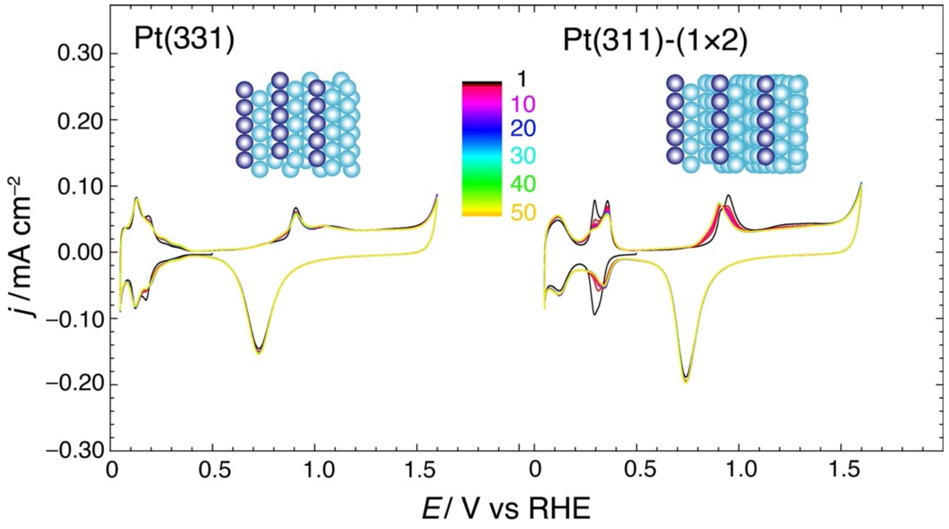

Figure S5. Cyclic voltammograms of Pt(331) -(1×1) and Pt(311)-(1×2) in 0.1 M $HClO_4$ between 0.05 and 1.6 V recorded over 50 potential cycles at scanning rate of 0.05 V s^{-1}.[1,2]

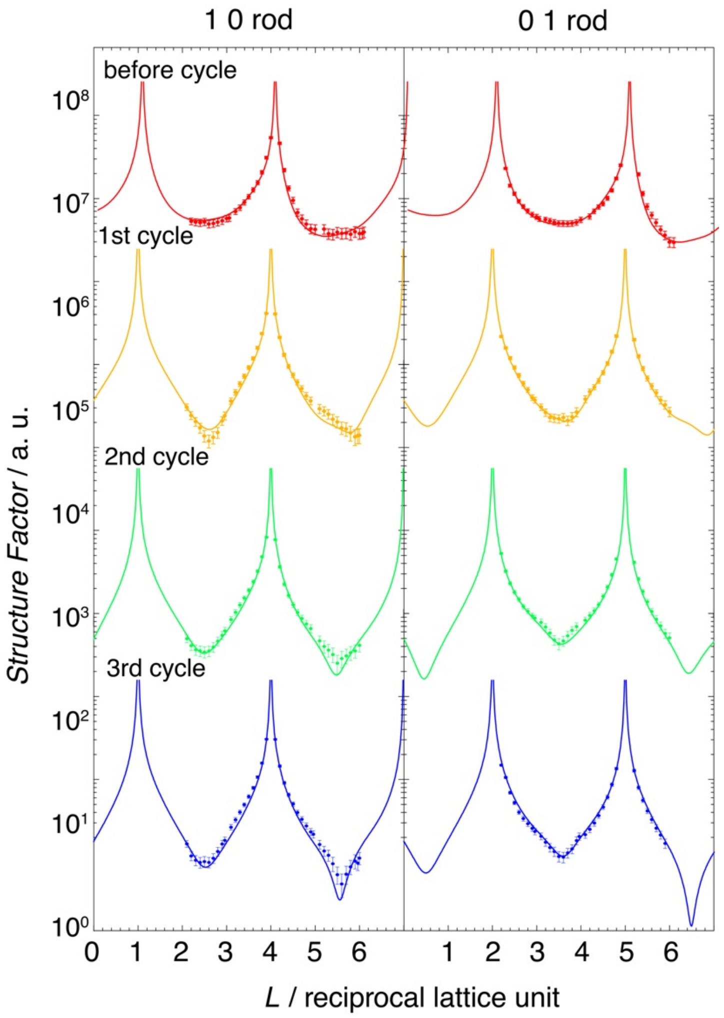

Figure S6. 10 and 01 CTRs of Pt(111) in 0.1 M $HClO_4$ at 1.0 V after each potential cycle up to 1.6 V. CTRs before the potential cycle was obtained at 0.9 V.

Table S1 | In plane and out of plane Debye Waller factors (*DW* / Å^2) and symmetrical reproducibility estimated from X-ray CTRs at 1.0 V after surface oxidation until 1.6 V.

		before (0.9 V)	1st (1.0 V)	2nd (1.0 V)	3rd (1.0 V)
In plane	*DW* (O_3)		2 ± 10	5 ± 16	2 ± 50
	DW (O_2)			2 ± 47	2 ± 30
	DW (O_1)	2 ± 10	50 ± 5	20 ± 5	20 ± 12

	DW (Pt$_4$)		22 ± 6	9 ± 4	14 ± 20
	DW (Pt$_3$)	1.2 ± 0.2	1.6 ± 0.3	0.5 ± 3	1.1 ± 0.3
	DW (Pt$_2$)	1.0 ± 0.3	0.4 ± 1.5	0.7 ± 0.3	0.5 ± 0.2
	DW (Pt$_1$)	0.5 ± 0.3	0.4 ± 0.2	0.9 ± 0.2	0.9 ± 0.2
Out of plane	DW (O$_3$)		2 ± 10	2 ± 8	1 ± 5
	DW (O$_2$)			1 ± 14	1 ± 14
	DW (O$_1$)	2 ± 2	4 ± 7	2 ± 2	4 ± 3
	DW (Pt$_4$)		7 ± 1	12 ± 2	12 ± 1
	DW (Pt$_3$)	1.1 ± 0.1	0.7 ± 0.1	0.5 ± 0.4	1.1 ± 0.2
	DW (Pt$_2$)	0.5 ± 0.1	0.9 ± 0.1	0.6 ± 0.1	0.5 ± 0.2
	DW (Pt$_1$)	0.5 ± 0.1	0.5 ± 0.1	0.7 ± 0.1	0.6 ± 0.1
symmetrical reproducibility		4.7 %	10.1 %	13.5 %	13.2 %

Supporting references

(1) Nakahara. A, Nakamura, M., Sumitani, K., Sakata, O. & Hoshi, N., In Situ Surface X-ray Scattering of Stepped Surface of Platinum: Pt(311). Langmuir, 23, 10879–10882 (2007).

DOI: 10.1021/la701566w

(2) Hoshi, N., Nakamura, M., Sakata, O., Nakahara, A., Naito, K. & Ogata, H., Surface X-ray scattering of stepped surfaces of platinum in an electrochemical environment: Pt (331)= 3 (111)-(111) and Pt (511)= 3 (100)-(111). Langmuir, 27, 4236–4242 (2011).

DOI: 10.1021/la200199b
