# Supplementary material for: Tailoring the active site for the oxygen evolution reaction on a Pt electrode
Source: Commun Chem. 2022 Oct 13;5:126. doi: 10.1038/s42004-022-00748-7 (PMC9814662; doi:10.1038/s42004-022-00748-7)
Supplement: Supplementary file 2 — Description of Additional Supplementary Data [file 42004_2022_748_MOESM2_ESM.docx]

Description of Additional Supplementary Files

**File name:** Supplementary Data 1

**Description:** Numerical data of Fig. 1

**File name:** Supplementary Data 2

**Description:** Numerical data of Fig. 2

**File name:** Supplementary Data 3

**Description:** Numerical data of Fig. 3

**File name:** Supplementary Data 4

**Description:** Numerical data of Fig. 4
